# Black soldier fly (*Hermetia illucens*) larvae oil as an alternative fat ingredient to soybean oil in laying hen diets

**DOI:** 10.5713/ab.22.0052

**Published:** 2022-05-07

**Authors:** Byeonghyeon Kim, Minji Kim, Jin Young Jeong, Hye Ran Kim, Sang Yun Ji, Hyunjung Jung, Seol Hwa Park

**Affiliations:** 1 Animal Nutrition and Physiology Division, National Institute of Animal Science, Rural Development Administration, Wanju 55365, Korea

**Keywords:** Egg Fatty Acid, Egg Quality, Heavy Metal, *Hermetia illucens*

## Abstract

**Objective:**

The objective of this study was to determine whether dietary black soldier fly (*Hermetia illucens*, HI) larvae oil (HILO) could serve as an alternative fat source to soybean oil (SBO) in laying hen diets.

**Methods:**

We randomly assigned 25-week-old Hy-line Brown laying hens (n = 144) to receive (n = 6 hens/group; eight replicates) a control or an experimental diet in which SBO was replaced with 50% (50HILO) or 100% HILO (100HILO).

**Results:**

Dietary HILO did not negatively affect body weight or productive performance during the study. The eggs also had similar quality parameters, proximate composition, and cholesterol levels. However, the yolk color index was significantly higher (p<0.01) in the 100HILO than in the other groups. Dietary HILO significantly altered the composition of fatty acids (FAs) in abdominal fat and eggs. Total saturated fatty acids (SFAs) and total polyunsaturated FAs (PUFAs) were significantly increased and decreased in the 50HILO and 100HILO groups, respectively, compared with those in the control group (p<0.001 and p<0.0001, respectively). Specifically, the medium-chain FAs lauric and myristic acids were remarkably increased in the abdominal fat of laying hens fed HILO (p<0.0001), whereas only myristic acid increased in eggs (p<0.0001). Undesirable heavy metal (aluminum, fluorine, arsenic, lead, mercury, and cadmium) concentrations were below permissible limits in eggs.

**Conclusion:**

We considered that HILO could be an alternative dietary fat to SBO for laying hens with maintained productive performance and good egg quality.

## INTRODUCTION

Soybean is a major protein and fat source for poultry diets, but the cost and limited supply have resulted in efforts to identify alternative sources of protein and fat for animal feed [1]. This is because the increasing demand of an expanding human population for animal protein will increase that for soybeans. Furthermore, managing organic waste from the increasing human population is a concern [1]. From this perspective, insects are potential feed ingredients for poultry diets [2] and the black soldier fly (*Hermetia illucens*, HI) is promising because it bioconverts organic waste into biomass [3,4], by storing protein and fat sources from organic waste decomposition during stage [5]. *Hermetia illucens* larvae (HIL) contain ~42% and 35% protein and fat sources, respectively [5,6]. Owing to its bioconversion and nutritional composition, HIL oil (HILO) has the potential as a fat source for chicken diets [7–9].

The nutrient composition in HIL can be altered by rearing conditions, such as feeding with substrates [10]. However, HIL typically have more abundant saturated fatty acids (SFA) than monounsaturated (MUFAs) and polyunsaturated (PUFAs) fatty acids [7,9,11]. The SFAs in HILO also contain more abundant lauric (C12:0) and myristic (C14:0) acids than those of other insect species, such as Argentinean cockroaches, house crickets, and yellow mealworms [12]. Lauric acid exerts functional antimicrobial effects on gut health in monogastric animals [13,14]. Furthermore, HILO has potential as a fat ingredient in broiler diets as it does not negatively impact health in terms of blood parameters and intestinal morphology [9,15]. Partially (50%) or completely (100%) replacing soybean oil (SBO) with HILO in broiler diets does not affect growth performance, carcass traits, meat quality, or health [7,9,11]. Cholesterol levels are also decreased in breast meat of chickens fed with HILO [11], but SFA content is increased. In contrast, PUFA content is decreased in breast and leg meat of broilers fed with HILO [7,11]. Eggs from laying quails fed with a diet containing 10% *H. illucens* larvae meal (HILM) had similarly higher SFA and MUFA contents than control eggs [16], and laying hen diets containing 4% HILM increase the MUFA and decreased the PUFA contents in eggs [17]. In contrast, others have found no changes in total SFA, MUFA, or PUFA contents in eggs from laying hens fed with 5% HILM [18]. A strategy to improve the fat composition of HIL by modulating its substrates or using HIL at an early stage of development when it has a high linoleic acid content might address the potential negative effects of HIL-derived meal and oil on the fatty acid (FA) profiles of animal products [11,12,19].

Although HILO is a promising fat ingredient for poultry, information about its inclusion in laying hen diets is limited. We also monitored the effects of heavy metal concentrations in eggs due to the potential risk of heavy metal accumulation in larvae bodies from contaminated organic waste [20]. In the present study, we replaced 50% or 100% of SBO with HILO as a dietary fat source in laying hen diets, and then evaluated the effects on productive performance, blood characteristics, body FA composition, quality, FA profile, and heavy metal concentrations of eggs.

## MATERIALS AND METHODS

### Insect oil, animals, and diets

The Institutional Animal Care and Use Committee of the Rural Development Administration approved this study (Approval No. NIAS-2020-498), which proceeded at the poultry facility of the National Institute of Animal Science of South Korea. Clean, dried HILs were press-defatted at 45°C to 48°C using an NF-80 cold press (Karaerler, Ankara, Turkey) to preserve nutritional and chemical quality.

A total of 144 Hy-Line Brown laying hens (age, 25 weeks; average live weight 1.93 kg±0.04 kg standard deviation [SD]) were randomly allocated to receive one of three diets (n = 48/group) and were housed in eight cages (n = 6/cage). The control group was fed with a corn-soybean meal-based diet, and the 50 and 100HILO groups were fed with diets in which SBO was partially (50%) or completely (100%) replaced with HILO, respectively. The diets were formulated to meet or exceed the requirements of the hens [21], and all groups were fed isoenergetic and isoproteic diets (Table 1). All hens had *ad libitum* access to feed and water during the trial.

### Productive performance

The hens (aged 25 weeks) were weighed at the beginning of the study. After adaptation to the experimental diets for 1 week, we counted the number of eggs produced to calculate the lay ratios (%) at the age of 26 to 33 weeks. Furthermore, the individual eggs were weighed daily to calculate the egg mass by multiplying the lay ratio (%) by egg weight. The feed conversion ratio (FCR) was calculated by dividing feed intake by egg mass.

### Egg quality determination

We randomly collected 48 eggs (n = 16/group: 2/cage) from cages at the end of the study and analyzed Haugh units (HU), yolk color, color, strength, and thickness of eggshells. We calculated HU as 100×log (albumen height – 1.7×egg weight^0.37^+7.6) as described by Eisen et al [22]. The colors of yolks and eggshells were measured using a Roche color fan (Hoffman-La Roche, Basel, Switzerland) and an eggshell color fan (Samyangsa Kangwon, Korea), respectively. Eggshell strength was determined using a TAHDi 500 texture analyzer (Stable Micro Systems, Godalming, UK), and eggshell thickness was measured at the top, middle, and bottom using a model 7360dial pipe gauge (Mitutoyo Corporation, Kawasaki, Japan).

### Chemical analyses of fat components and eggs

Three eggs per cage were homogenized (eight replicates/group) to analyze proximate composition according to the AOAC procedure [23], cholesterol levels using gas chromatography (GC) as described [24], and FA profiles as described [17]. Total lipids were determined as described by Folch et al [25], and the methyl esters of lipids were measured using a Star 3600 GC instrument (Varian Technologies, Palo Alto, CA, USA) with an Omegawax 205 Fused Silica Capillary GC Column (30 m×0.32 mm×0.25 μm film thickness). The temperatures of the injection port and detector were 250°C and 260°C, respectively. The results are presented as ratios (%) of total FA.

### Heavy metal determination

Heavy metal contamination was assessed in eggs after chemical analyses. Homogenized egg samples were digested with nitric acid and hydrogen peroxide using a microwave. Then heavy metal concentrations were quantified using an Agilent 7700x inductively coupled plasma-mass spectrometer (Agilent Technologies, Santa Clara, CA, USA).

### Statistical analysis

Each cage was considered as an experimental unit for productive performance and physical quality of eggs, whereas the experimental unit for proximate composition comprised egg homogenates, FA profiles, and heavy metal concentrations. Data were statistically analyzed using PROC general linear models (GLM) of the Statistical Analysis System (SAS; SAS Institute Inc., Cary, NC, USA), and means were compared using Tukey’s multiple comparison test [26]. Values with p<0.05 and p<0.10 were considered as being significant and having a tendency, respectively.

## RESULTS

### Fatty acid profiles of larvae and SBO

Table 2 summarizes the FA composition of HILO and SBO. The SFA content was higher in HILO than in SBO (61.36% vs 12.09%). The proportions of lauric and myristic acids were higher in the HILO than in SBO (41.24% vs 0.12% and 5.61% vs 0.26%, respectively). In contrast, total PUFA was higher in SBO than HILO (65.74% vs 15.58%).

### Body weight and productive performance

Tables 3 and 4 show the effects of HILO on the body weight and productive performance of laying hens. The initial and final body weight did not significantly differ between the HILO and control groups. In addition, HILO did not negatively affect parameters of productive performance, namely, lay percentage, egg weight and mass, feed intake, and FCR.

### Physical quality and chemical composition of eggs

Tables 5 and 6 show the effects of dietary HILO on physical traits, proximate composition, and cholesterol levels in eggs. Yolk height, HU, strength, thickness, and eggshell color did not significantly differ from controls. However, HILO increased the intensity of the yolk color (p = 0.004). Furthermore, water, protein, lipid, ash, or cholesterol levels did not significantly differ among the groups.

### Fatty acid composition of abdominal fat and eggs

Table 7 shows the FA profile of abdominal fat in laying hens. The SFAs, capric, lauric, and myristic acids, were significantly increased (p<0.0001), whereas arachidic acid was decreased (p = 0.0247) in the 50HILO and 100HILO groups compared with that in the controls. Total MUFA content was not altered, but myristoleic and palmitoleic acids were increased (p<0.01) in the 50HILO, compared with that in the control group. In contrast, total PUFA, including linoleic and linolenic acid, as well as UFA/SFA, *n*-6 FA, and *n*-3 FA levels were significantly lower in the HILO than in the control group (p<0.0001 for all). However, *n*-6/*n*-3 was increased (p = 0.0002) in the 100HILO compared with that in the control and 50HILO groups.

The FA composition in the eggs was similar (Table 8). Total SFA and PUFA were respectively increased (p = 0.0002) and decreased (p<0.0001) in the 50HILO and 100HILO eggs compared with that in the controls. Palmitic acid was significantly increased (p = 0.0211) by HILO. Linoleic and linolenic acids among PUFAs were significantly lower in the 50HILO and 100HILO (p<0.0001) eggs than in the controls. The contents of UFA/SFA and *n*-6 FA were decreased (p< 0.001), whereas those of *n*-6/*n*-3 was decreased (p = 0.0009) in the 100HILO, compared with those in the control group.

### Heavy metal concentrations in eggs

Table 9 shows the heavy metal concentrations in eggs of laying hens fed with HILO. Among essential elements, the concentration of sulfur was decreased (p<0.0001) in both HILO groups compared with that in the controls. Essential and trace elements in the eggs from laying hens fed with HILO were not applicable for restricting permissible limits. The aluminum (Al) concentration was increased (p = 0.037) in the 100HILO compared with that in the control group, and was otherwise undetectable in the 50HILO group. The levels of fluorine (F), arsenic (As), lead (Pb), mercury (Hg), and cadmium (Cd) were below the limits of detection in all groups.

## DISCUSSION

The weight of laying hens positively correlates with egg size and is thus important for productive performance [27]. We found that replacing SBO with HILO did not alter body weight throughout the study. To the best of our knowledge, the effect of dietary HILO on the weight of laying hens has remained unknown. The growth performance of broiler chickens is not affected by dietary HILO [7,9]. The weight did not significantly differ among the three groups. However, HILM negatively affects productive performance in terms of lay percentage, feed intake, egg mass, and FCR in laying hens [17,28,29]. The decreased feed intake in laying hens fed with HILM can be explained by the dark color of HILM [8,28]. Furthermore, smell differences due to lipid oxidation caused by processing HILO at high temperatures can decrease feed intake in laying hens [8]. We extracted oil from dried larvae using a cold press, which has the advantages of preventing oxidation reactions, denaturation, and nutritional loss caused by heat [17,30]. Hence, cold-pressed HILO is a suitable fat ingredient in laying hen diets as it does not affect productive performance.

We found no adverse effects on egg quality. These results substantiate previous findings in which HILO did not affect yolk height, HU, strength, thickness, or eggshell color [8]. However, increasing the concentration of HILO in laying hen diets intensified yolk color, which was also in line with the result of a study where laying hens were fed with HILM and HILO prepared from larvae reared on various substrates and processed differently. The HILM had higher ether extract (EE) levels than the former HILM (299 vs 133 g/kg). Furthermore, laying hens that consumed diets with total soybean cake and SBO replacement with HILM and HILO as dietary protein and fat sources laid eggs with intense red egg yolks. Carotenoid absorption might have been increased due to a higher EE content in the HILM in that study than that in previous studies [8]. Feeding laying hens and quails with 10% and 15% HILM increased the yolk color index [16,31]. Furthermore, 4% HILM in laying hen diets increases [17], whereas 1%, 3%, and 5% HILM decrease the yolk index [18]. Although the reason for this is unclear, the discrepancies in the results could be ascribed to other dietary components, such as the ratio (%) of corn. The authors formulated experimental diets with less corn to substitute soybean meal with HILM compared with the control diet [18], unlike others [16,17,31]. Hence, an increased amount of corn could affect the yolk color due to its carotenoid content [32]. Here, the EE content and corn ratios of all experimental diets were similar; therefore, pigments in carotenoids such as lutein, zeaxanthin, and β-carotene in the HILM and HILO can help to improve yolk color [16,33].

We found that adding HILO to laying hen diets did not alter the proximate composition and cholesterol content of eggs. The effects of HILM on the chemical composition of eggs have been reported [16,33], but only a few studies have investigated the effects of HILO in laying hen diets. Heuel et al [8] found no alterations in the EE content of egg yolk from laying hens fed with HILO; they explained that the reason is ascribed to similar EE contents in diets among treatments. They also reported that the high EE content in the HILM diet contributed to increased EE content in egg yolks [8]. The EE contents of the experimental diets were similar to the present study; therefore, the EE content in eggs was not altered. Our findings are in line with those of a study showing that a similar EE content in a HILM-based diet did not affect the EE content in egg yolk [33].

The effects of dietary HILM and HILO on cholesterol levels in eggs and meat have been investigated [11,16,33]. The cholesterol content was decreased in eggs of laying hens fed HILM, and chitin might have contributed [17,33]. This notion is supported by the finding that chitin can contribute to decreased lipid absorption and thus decrease blood levels of cholesterol [28,34]. Furthermore, the chitin derivative, chitosan, increases bile acid and fat excretion in rat feces, which might also reduce cholesterol absorption [35]. To the best of our knowledge, little is known about the effects of HILO on cholesterol levels in eggs. However, replacing SBO with 50% HILO decreases cholesterol levels in chicken breast meat [11]. In fact, medium-chain fatty acids (MCFAs) can increase serum levels of cholesterol and high-density lipoprotein cholesterol [15,36]. However, cholesterol levels in eggs did not differ among the control, 50HILO, and 100HILO groups in the present study, although HILO has a high MCFA content. Our results agree with those of a study suggesting that broilers differently metabolize MCFAs in HILO and MCFA-rich coconut oil [15]. Thus, we assumed that MCFA in HILO cannot alter cholesterol levels compared with HILM and coconut oil in the absence of chitin and the nature of lipid metabolism.

The FA composition of abdominal fat and eggs reflected that of HILO. The effects of HILM and HILO on FA profiles in abdominal fat and animal products such as meat and eggs have been investigated [7,15,18,37]. The SFA content is the predominant FA in HIL-derived meal and oil [11,15,37]. We found that total SFA and PUFA contents were respectively increased and decreased in the abdominal fat of hens and in eggs. In addition, the altered FA composition of abdominal fat caused by HILO did not change with age in broilers [15]. The present study lasted 9 weeks and we found that dietary HILO has lasting effects on the FA composition of abdominal fat and eggs. The MCFAs, capric, lauric, and myristic acids were significantly increased in the abdominal fat of chickens fed HILO [15,38], but to a lesser extent in eggs than abdominal fat. This could be explained by the fact that an altered FA composition is more pronounced in adipose tissues than in animal products because of physiological lipid storage [39,40]. Furthermore, changes in the FA profile of HILO eggs might be considered undesirable in terms of human health. From this perspective, the FA composition of HIL could be improved by modulating their rearing substrate with a high content of PUFA [10] or by using HIL at an early stage of development [19]. The level of linolenic acid is maximal (31.4%) in 6-day-larvae and that of lauric acid is minimal (7.6%) in 4-day-larvae [19]. Thus using early-stage HIL might be a useful strategy for increasing the PUFA content in the HILO.

We assessed the concentrations of heavy metals in eggs to avoid potential health risks for consumers. Heavy metal concentrations in larvae can be increased by dietary consumption during the growth stages of larvae, prepupae, and adult black soldier flies [20,41]. Even though the heavy metal concentration was higher in larvae than in prepupae by defecation before emergence, undesirable substances in HILM and animal products (such as chicken breast meat and eggs) from animals fed with HILM were below permissible limits, as we previously showed [17,20,42]. Likewise, concentrations of undesirable heavy metals such as Al, F, As, Pb, Hg, and Cd in eggs were acceptable in the present study; therefore, HILO might be a suitable fat ingredient for laying hen diets in terms of safety.

## CONCLUSION

Total replacement of SBO with HILO in laying hen diet from 25 to 33 weeks of age did not adversely affect productive performance, egg quality, nutritional composition, or cholesterol levels in eggs. The inclusion of HILO increased the egg yolk color index and MCFA profiles in abdominal fat. Taken together, our findings suggest that HILO can be used as a fat ingredient in laying hen diets. This should increase consumer acceptance of eggs by intensifying yolk color without safety concerns regarding heavy metal concentrations. However, further studies are needed to improve the FA composition of eggs by modulating feeding substrates or using early-stage HIL.

## Figures and Tables

**Table 1:** Composition (%) of the experimental laying hens diets containing different levels of *Hermetia illucens* larvae oil (HILO)

Item	Dietary treatments^1)^		

	CON	50HILO	100HILO
Ingredients (%)			
Corn	61.03	61.03	61.03
Soybean meal, 45%	23.70	23.70	23.70
Wheat bran	2.00	2.00	2.00
Soybean oil	3.00	1.50	0.00
HILO	0.00	1.50	3.00
Dicalcium phosphate	1.04	1.04	1.04
Limestone	8.56	8.56	8.56
L-lysine, 78%	0.02	0.02	0.02
DL-methionine	0.15	0.15	0.15
Salt	0.20	0.20	0.20
Vitamin-mineral premix^2)^	0.30	0.30	0.30
Calculated composition			
ME (kcal/kg)	2,765	2,750	2,735
Lysine	0.83	0.83	0.83
Methionine	0.41	0.41	0.41
Calcium	3.80	3.80	3.80
Total phosphorus	0.60	0.60	0.60
Analyzed composition			
Crude protein	15.96	16.11	15.99
Crude fat	5.90	5.91	5.82
NDF	8.50	8.93	8.96
ADF	3.14	3.28	3.37
Ash	9.58	11.05	10.93

ME, metabolizable energy; NDF, neutral detergent fiber; ADF, acid detergent fiber.

1) CON, control diet; 50 and 100HILO, HILO groups in which the soybean oil was replaced with 50% and 100% of the HILO, respectively.

2) Supplied per kilogram of diet: vitamin A 8,000 IU; vitamin D_3_ 3,300 IU; vitamin E 80 mg; vitamin K_3_ 3 mg; vitamin B_1_ 3 mg; vitamin B_2_ 8 mg; vitamin B_6_ 6 mg; vitamin B_12_ 0.04 mg; nicotinic acid 50 mg; pantothenic acid 16 mg; folic acid 2 mg; choline chloride 600 mg; Mn 90 mg; Zn 85 mg; Fe 70 mg; Cu 10 mg; I 1.8 mg; Co 0.6 mg; Se 0.35 mg.

**Table 2:** Fatty acid profile (% of total fatty acid methyl esters) of the *Hermetia illucens* larvae oil (HILO) and soybean oil

Item	HILO	Soybean oil
Fatty acids		
C10:0 (Capric)	2.07	0.01
C12:0 (Lauric)	41.24	0.12
C14:0 (Myristic)	5.61	0.26
C16:0 (Palmitic)	12.42	7.38
C17:0 (Magaric)	0.13	0.15
C18:0 (Stearic)	1.96	4.17
SFA	61.36	12.09
C14:1 (Myristoleic)	0.11	0.06
C16:1 (Palmitoleic)	2.49	0.62
C18:1 (Oleic)	14.72	19.38
C20:1n-9 (Eicosenoic)	1.65	0.18
MUFA	19.13	20.29
C18:2n-6 (Linoleic)	14.02	54.82
C18:3n-3 (Linolenic)	0.58	10.21
C20:2n-6 (Eicosadienoic)	0.06	0.08
C20:5n-3 (Eicosapentaenoic)	0.74	0.51
PUFA	15.58	65.74
SFA/UFA	1.77	0.14
*n*-6	14.08	54.90
*n*-3	1.50	10.84
*n*-6/*n*-3	9.39	5.06

SFA, saturated fatty acid; MUFA, monounsaturated fatty acid; PUFA, polyunsaturated fatty acid; UFA, unsaturated fatty acid.

**Table 3:** Changes in body weight (BW) of laying hens during the trial

Item	Dietary treatments^1)^			SEM	p-value

	CON	50HILO	100HILO		
Initial BW (g)	1,931.99	1,922.69	1,923.41	15.37	0.896
Final BW (g)	2,013.47	2,007.85	1,979.17	29.07	0.708
Weight gain (g)	81.48	85.17	55.76	21.51	0.623

SEM, standard error of the means.

1) CON, control diet; 50 and 100HILO, HILO groups in which the soybean oil was replaced with 50% and 100% of the HILO, respectively.

**Table 4:** Effect of the dietary *Hermetia illucens* larvae oil (HILO) inclusion level on the productive performance of laying hens

Item	Dietary treatments^1)^			SEM	p-value

	CON	50HILO	100HILO		
Lay (%)	96.17	97.58	96.61	1.24	0.716
Egg weight (g)	60.41	59.82	60.80	0.42	0.267
Feed intake (g/d/hen)	121.28	121.79	126.20	1.79	0.126
Egg mass	58.03	58.34	58.74	0.73	0.793
FCR	2.09	2.09	2.15	0.03	0.220

SEM, standard error of the means; FCR, feed conversion ratio.

1) CON, control diet; 50 and 100HILO, HILO groups in which the soybean oil was replaced with 50% and 100% of the HILO, respectively.

**Table 5:** Effect of the dietary *Hermitia illucens* larvae oil (HILO) inclusion level on egg quality

Physical attribute	Dietary treatments^1)^			SEM	p-value

	CON	50HILO	100HILO		
Haugh unit	91.58	92.77	88.78	1.51	0.183
Eggshell strength (kgf/cm^2^)	5.00	4.78	5.16	0.35	0.742
Eggshell thickness (mm)	0.43	0.42	0.42	0.01	0.476
Eggshell color (fan)	12.94	13.50	13.19	0.39	0.602
Yolk color (fan)	6.31^b^	6.25^b^	7.13^a^	0.18	0.004

SEM, standard error of the means.

1) CON, control diet; 50 and 100HILO, HILO groups in which the soybean oil was replaced with 50% and 100% of the HILO, respectively.

a,b Values with different superscripts in the same row are significantly different (p<0.01).

**Table 6:** Effect of the dietary *Hermitia illucens* larvae oil (HILO) inclusion level on proximate composition and cholesterol content of eggs

Item	Dietary treatments^1)^			SEM	p-value

	CON	50HILO	100HILO		
Water (%)	76.45	76.53	76.22	0.31	0.662
Protein (%)	12.68	12.48	12.79	0.13	0.190
Lipids (%)	9.27	9.24	9.29	0.16	0.981
Ash (%)	0.92	0.90	0.93	0.01	0.164
Cholesterol (mg/100 g)	344.02	341.99	353.71	6.25	0.160

SEM, standard error of the means.

1) CON, control diet; 50 and 100HILO, HILO groups in which the soybean oil was replaced with 50% and 100% of the HILO, respectively.

**Table 7:** Effect of the dietary *Hermetia illucens* larvae oil (HILO) inclusion level on the fatty acid profile (% of total fatty acid methyl esters) of abdominal fat in laying hens

Item	Dietary treatments^1)^			SEM	p-value

	CON	50HILO	100HILO		
Fatty acids					
C10:0 (Capric)	0.00^c^	0.04^b^	0.08^a^	0.01	<0.0001
C12:0 (Lauric)	0.10^c^	2.23^b^	4.39^a^	0.28	<0.0001
C14:0 (Myristic)	0.53^c^	1.25^b^	1.77^a^	0.07	<0.0001
C16:0 (Palmitic)	21.44	23.51	22.61	0.63	0.0873
C18:0 (Stearic)	6.69	6.77	6.71	0.20	0.9584
C20:0 (Arachidic)	0.11^a^	0.09^b^	0.09^b^	0.01	0.0247
Total SFA	29.15^b^	34.17^a^	35.95^a^	0.75	<0.0001
C14:1 (Myristoleic)	0.07^b^	0.15^a^	0.18^a^	0.02	0.0002
C15:1 (Pentadecenoic)	0.03	0.03	0.03	0.01	0.9864
C16:1 (Palmitoleic)	2.21^b^	3.16^a^	2.89^ab^	0.20	0.0082
C18:1 (Oleic)	37.93	38.09	38.07	0.64	0.9833
C20:1 n-9 (Eicosenoic)	0.26	0.25	0.28	0.01	0.0732
Total MUFA	40.63	41.77	41.57	0.70	0.4859
C18:2 n-6 (Linoleic)	28.47^a^	22.52^b^	21.29^b^	0.98	<0.0001
C18:3 n-6 (γ-Linolenic)	0.11	0.10	0.10	0.01	0.5881
C18:3 n-3 (Linolenic)	1.37^a^	1.07^b^	0.78^c^	0.05	<0.0001
C20:4 n-6 (Arachidonic)	0.06	0.07	0.08	0.01	0.1378
C20:5 n-3 (Eicosapentaenoic)	0.05	0.05	0.03	0.01	0.1962
C22:2 n-6 (Docosadienoic)	0.11	0.21	0.15	0.03	0.1391
Total PUFA	30.23^a^	24.06^b^	22.48^b^	1.01	<0.0001
UFA/SFA	2.45^a^	1.93^b^	1.79^b^	0.07	<0.0001
*n*-6	28.81^a^	22.95^b^	21.67^b^	0.98	<0.0001
*n*-3	1.42^a^	1.12^b^	0.81^c^	0.05	<0.0001
*n*-6/*n*-3	20.46^b^	20.67^b^	27.11^a^	1.06	0.0002

SEM, standard error of the means; SFA, saturated fatty acid; MUFA, monounsaturated fatty acid; PUFA, polyunsaturated fatty acid; UFA, unsaturated fatty acid.

1) CON, control diet; 50 and 100HILO, HILO groups in which the soybean oil was replaced with 50% and 100% of the HILO, respectively.

a–c Values with different superscripts in the same row are significantly different (p<0.05).

**Table 8:** Effect of the dietary *Hermetia illucens* larvae oil (HILO) inclusion level on the fatty acid profile (% of total fatty acid methyl esters) of laying hen eggs

Item	Dietary treatments^1)^			SEM	p-value

	CON	50HILO	100HILO		
Fatty acids					
C12:0 (Lauric)	0.18	0.13	0.24	0.04	0.2170
C14:0 (Myristic)	0.34^c^	0.96^b^	1.67^a^	0.06	<0.0001
C16:0 (Palmitic)	25.48^b^	27.12^a^	27.15^a^	0.44	0.0211
C17:0 (Magaric)	0.20	0.20	0.18	0.01	0.2469
C18:0 (Stearic)	9.34	9.99	9.29	0.25	0.1184
C20:0 (Arachidic)	0.03	0.06	0.04	0.01	0.2732
C21:0 (Heneicosylic)	0.13	0.15	0.14	0.01	0.1564
C24:0 (Lignoceric)	0.12^b^	0.15^a^	0.14^ab^	0.01	0.0334
Total SFA	35.87^b^	38.81^a^	38.91^a^	0.48	0.0002
C14:1 (Myristoleic)	0.05^c^	0.16^b^	0.34^a^	0.02	<0.0001
C16:1 (Palmitoleic)	2.09^b^	2.46^b^	3.01^a^	0.12	0.0001
C18:1 (Oleic)	39.93	38.78	39.76	0.48	0.2138
C20:1 n-9 (Eicosenoic)	0.17	0.16	0.18	0.01	0.5179
Total MUFA	42.38	41.74	43.48	0.49	0.0620
C18:2 *n*-6 (Linoleic)	18.17^a^	15.91^b^	13.97^c^	0.48	<0.0001
C18:3 *n*-6 (γ-Linolenic)	0.13	0.14	0.12	0.01	0.0549
C18:3 *n*-3 (Linolenic)	0.58^a^	0.43^b^	0.35^c^	0.02	<0.0001
C20:2 *n*-6 (Eicosadienoic)	0.15^a^	0.14^a^	0.11^b^	0.01	0.0016
C20:4 *n*-6 (Arachidonic)	1.76	1.88	1.99	0.12	0.4487
C20:5 *n*-3 (Eicosapentaenoic)	0.01^b^	0.03^b^	0.05^a^	0.01	0.0005
C22:2 *n*-6 (Docosadienoic)	0.28	0.13	0.15	0.06	0.1572
C22:6 *n*-3 (Docosahexaenoic)	0.69	0.80	0.88	0.06	0.1194
Total PUFA	21.76^a^	19.46^b^	17.64^b^	0.53	<0.0001
UFA/SFA	1.79^a^	1.58^b^	1.57^b^	0.03	0.0001
*n*-6	20.48^a^	18.19^b^	16.33^c^	0.49	<0.0001
*n*-3	1.28	1.26	1.28	0.07	0.9762
*n*-6/*n-*3	16.09^a^	14.45^ab^	13.04^b^	0.48	0.0009

SEM, standard error of the means; SFA, saturated fatty acid; MUFA, monounsaturated fatty acid; PUFA, polyunsaturated fatty acid; UFA, unsaturated fatty acid.

1) CON, control diet; 50 and 100HILO, HILO groups in which the soybean oil was replaced with 50% and 100% of the HILO, respectively.

a–c Values with different superscripts in the same row are significantly different (p<0.05).

**Table 9:** Heavy metal concentrations (mg/kg) in eggs of laying hens fed *Hermetia illucens* larvae oil (HILO)

Item	Dietary treatments^1)^			SEM	p-value	Permissible limit

	CON	50HILO	100HILO			
Essential elements						
Mg	122.16	116.65	117.17	1.65	0.052	NA
S	2,137.50^a^	2,000.00^b^	2,037.50^b^	18.50	<0.0001	NA
Essential trace elements						
Fe	22.22	20.97	21.65	0.58	0.327	NA
Zn	15.79	15.40	15.56	1.09	0.968	NA
Cu	<0.10	<0.10	<0.10	-	-	NA
Cr	<0.10	<0.10	<0.10	-	-	NA
Co	<0.10	<0.10	<0.10	-	-	NA
Se	<0.10	<0.10	<0.10	-	-	NA
Mn	<0.10	<0.10	<0.10	-	-	NA
I	ND	ND	ND	-	-	NA
Undesirable elements						
Al	2.21^b^	2.87^ab^	3.66^a^	0.37	0.037	30.00 [43]
F	<0.01	<0.01	<0.01	-	-	NA
As	<0.01	<0.01	<0.01	-	-	0.04 [44]
Pb	<0.01	<0.01	<0.01	-	-	0.20 [45]
Hg	<0.01	<0.01	<0.01	-	-	0.05 [45]
Cd	<0.10	<0.10	<0.10	-	-	0.05 [45]

SEM, standard error of the means; NA, not applicable; ND, not detected.

1) CON, control diet; 50 and 100HILO, HILO groups in which the soybean oil was replaced with 50% and 100% of the HILO, respectively.

a,b Values with different superscripts in the same row are significantly different (p<0.05).
